# Human cornea thermo-viscoelastic behavior modelling using standard linear solid model

**DOI:** 10.1186/s12886-023-02985-3

**Published:** 2023-06-05

**Authors:** Hassan M. Ahmed, Nancy M. Salem, Walid Al-Atabany

**Affiliations:** 1 Biomedical Engineering Department, Helwan University, Helwan, Egypt; 2 Information Technology and Computer Science School, Nile University, Sheikh Zayed City, Egypt

**Keywords:** Corneal viscoelasticity, Corneal modelling, Corneal biomechanics, Corneal thermal behavior

## Abstract

**Background:**

Corneal biomechanics is of great interest to researchers recently. Clinical findings relate them to corneal diseases and to outcomes of refractive surgery. To have a solid understanding of corneal diseases’ progression, it is important to understand corneal biomechanics. Also, they are essential for better explaining outcomes of refractive surgeries and their undesired consequences. There is a difficulty for studying corneal biomechanics in-vivo and multiple limitations arise for ex-vivo studies. Hence mathematical modelling is considered as a proper solution to overcome such obstacles. Mathematical modelling of cornea in-vivo allows studying corneal viscoelasticity with taking into consideration all boundary conditions existing in real in-vivo situation.

**Methods:**

Three mathematical models are used to simulate corneal viscoelasticity and thermal behavior in two different loading situations: constant and transient loading. Two models of the three are used for viscoelasticity simulation which are Kelvin-Voigt and standard linear solid models. Also, temperature rise due to the ultrasound pressure push is calculated using bioheat transfer model for both the axial direction and as a 2D spatial map using the third model (standard linear solid model).

**Results:**

Viscoelasticity simulation results show that standard linear solid model is efficient for describing the viscoelastic behavior of human cornea in both loading conditions. Results show also that the deformation amplitude obtained from standard linear solid model is more reasonable for corneal soft-tissue deformation with respect to corresponding clinical findings than that obtained from Kelvin-Voigt model. Thermal behavior results estimated corneal temperature rise to be roughly 0.2 °C, which conforms with FDA regulations for soft tissue safety.

**Conclusion:**

Standard Linear Solid (SLS) model is better describing the human corneal behavior in response to constant and transient load more efficiently. Temperature rise (TR) for the corneal tissue of about 0.2 °C is conforming with FDA regulations and even less than the FDA regulations for soft tissue safety.

## Introduction

Deformation behavior of soft tissues due to stress is investigated by measuring their biomechanics properties. Recently, research is giving attention to biomechanical properties of ocular tissues so as to understand the progression of ocular diseases such as: keratoconus, and post-refractive ectasia which are characterized by corneal tissue deformation. Also, elasticity changes before and after the refractive surgeries are of their interest as they affect the developing of post-refractive ectasia. Biomechanical properties are reported to be changing due to keratoconous corneas and refractive surgeries [1], [2]. A comprehensive review about the impact of post-refractive surgeries on corneal biomechanics and how can they be measured is presented by our previous paper [3].

Understanding of cornea biomechanics leads to better understanding of these corneal alterations and how to treat them. Many researches have focused on investigation of the corneal biomechanics ex-vivo such as the work reported in [4]. However, ex-vivo cornea experience swelling and loss of its tear film leading to changes in its biomechanical properties [1]. Hence, there is a great need for in-vivo cornea studies that allows for normal and diseased cornea biomechanics. Mathematical modelling of corneal biomechanics is the first step in understanding its behavior in normal and diseased states. A suitable model should describe the elastic and viscous components of the cornea as its tissue exhibits viscoelastic behavior when loaded under certain forces transiently [2].

Human cornea experiences a viscoelastic behavior when subjected to a transient stress [5]. This behavior can be represented by two components; elastic component and viscous component. The elastic component gives an instantaneous deformation while the viscous component gives a damping deformation. Elastic and viscous components can be modelled by spring; with elasticity of (E); and dashpot; with viscosity of ($\eta$); system components respectively. The spring represents the pure elastic behavior to an applied load, while the dashpot represents the time-dependent viscous resistance to that load.

**Fig. 1:**
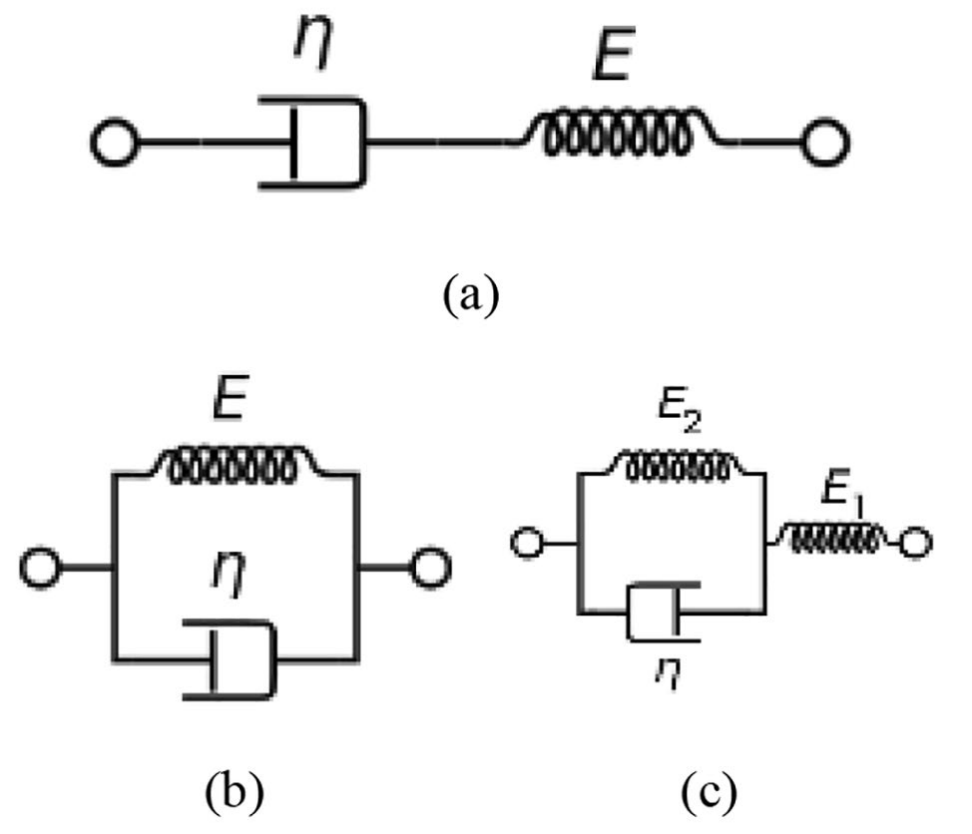
(**a**) Maxwell Model, (**b**) Kelvin-Voigt and (**c**) Standard linear solid model in Kelvin-Voigt representation

There are multiple models that are used to describe the viscoelasticity behavior, amongst them Maxwell model, Kelvin-Voigt model and the standard linear solid model [6]. The three models are presented in Fig. 1. Maxwell’s model consists of a spring and a dashpot connected in series to each other, while Kelvin-Voigt model consists of the same two components but connected in parallel to each other. Standard linear solid model in Kelvin-Voigt representation has an extra spring element connected in series to the parallel spring-dashpot connection.

Several research proposed mathematical models for studying corneal biomechanics. One of them is the model reported in [5], where two Kelvin-Voigt models in series with a spring is proposed to compare the effect of fast versus slow viscoelastic behavior. In [7], a Kelvin-Voigt model is proposed to study the effect of elasticity and viscosity separately on corneal hysteresis. In [8], a mathematical model based simulation is proposed to investigate induced corneal vibrations due to air-puff and its relationship to intra-ocular pressure and viscoelasticity of the cornea. Other mathematical models are proposed to study the heating effect of different wavelengths of UV lasers used in corneal ablation procedures as well [9–11]. Temperature rise calculation due to microwave radiation formed the first step towards modelling the thermal behavior of the human eye [12], [13]. While the first FEM model developed for describing the human eye thermal behavior while undergoing LASER radiation is introduced by [14].

This paper presents a mathematical model to investigate the corneal tissue viscoelastic behavior and thermal behavior, i.e., temperature rise (TR); when subjected to a transient force under the effect of intra-ocular pressure and tear film pressure. This study represents a continuation of our project that is presented in [15].

The paper is organized as follows, Sect. 1 presents an introduction about the importance of corneal biomechanics to corneal diseases and refractive surgeries outcomes, different mathematical models that are used to describe soft-tissue biomechanics and thermal behavior and advantages of each one with respect to the others. The proposed methodology for the simulation procedure using both models is introduced in Sect. 2. Results are presented and discussed in Sect. 3 and the paper is concluded in Sect. 4.

## Methodology

The viscoelastic and the thermal behaviors of the corneal tissue are simulated using mathematical equations to investigate the corneal behavior in response to ultrasonic transient load. The viscoelastic behavior is simulated using a constant and a transient load simulation. While the thermal behavior is simulated in case of a transient load only. The methodology is divided into two major sections, one for simulating the viscoelastic behavior and the other for simulating the thermal behavior. The first major section is furthermore divided into two subsections, one for simulating the corneal viscoelastic behavior due to constant load and the other for simulating its behavior due to the transient load. Afterwards; the corneal viscoelastic behavior is simulated for a wide range of Young’s moduli starting from 150 KPa till 3 MPa [16], [17] in order to predict whether the corneal behavior is linear or not. This is in correspondence with Fung et *al.* [18], [19] and Hoeltzel et *al.* [20], where they recommend that for nonlinear behavior tissues, the study should not be carried out for a single Young’s modulus.

### Viscoelastic behavior

In this section, both the Kelvin-Voigt and the standard linear solid models are used to simulate the human cornea viscoelastic behavior. Maxwell’s model is discarded due to its indefinite creep behavior and because it does not fully recover from deformation due to the pure viscous component.

**Fig. 2:**
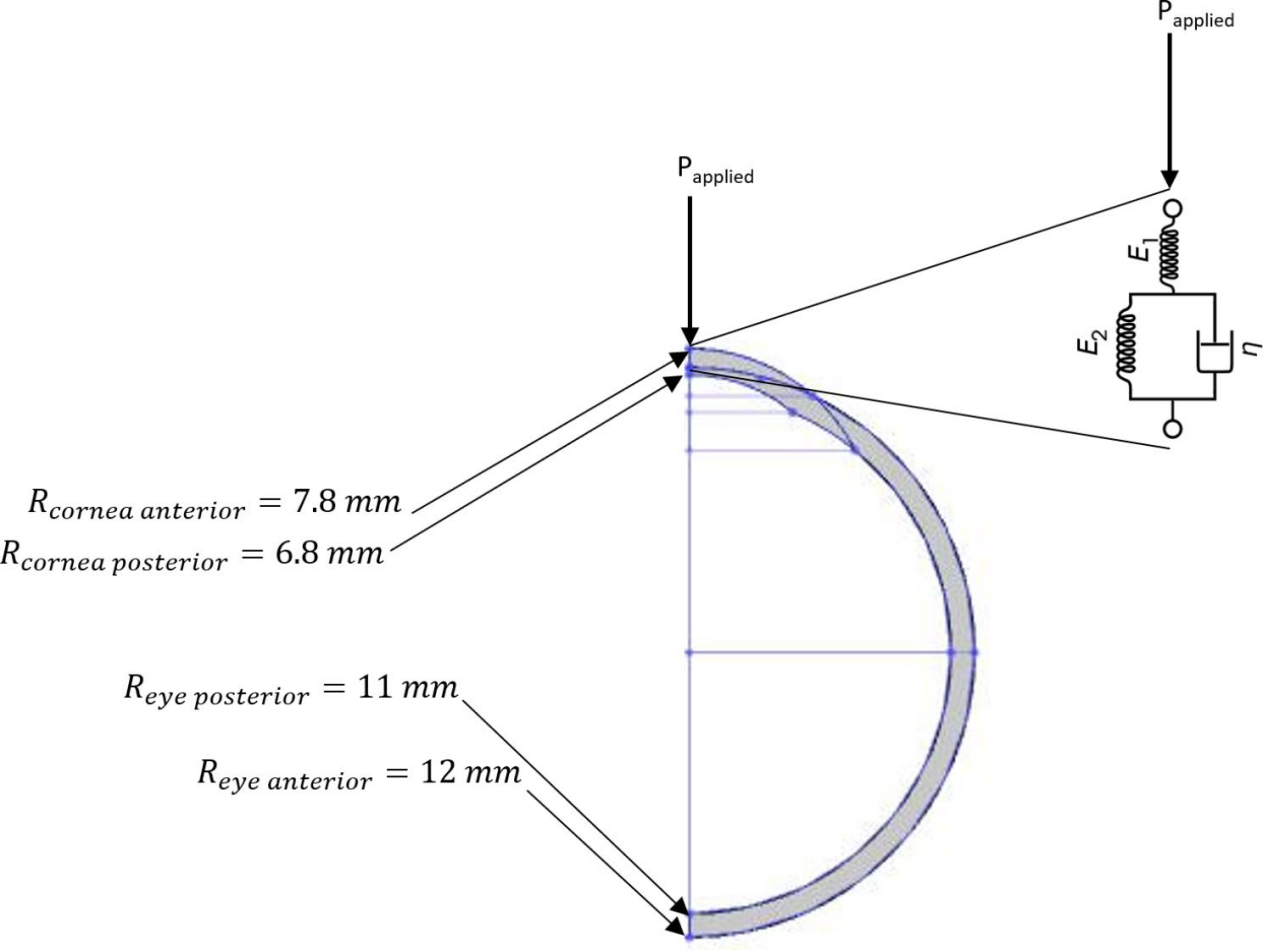
Complete vertical cross-section of human eye, and corneal tissue represented with standard linear solid model

The complete vertical cross-section of human eye with cornea represented as standard linear solid model is shown in Fig. 2. The corneal tissue is modelled using Kelvin-Voigt model and standard linear solid model. Its behavior using both models is simulated under two scenarios; while applying a constant load and a transient load respectively. The constant load is simulated as a unit step function, and the transient load is simulated as a sinusoidal burst of 3 cycles applied for 40% of the simulation time. The transient load is chosen to be a sinusoidal burst as it simulates the acoustic radiation force burst as an internal actuator generated from an ultrasound transducer during elastography procedure [3], [15], [21].

The corneal tissue elasticity is set to 150 KPa to simulate a normal corneal tissue [22]. Either the constant load or the transient load is applied at the corneal apex. The simulation time is 5 msec for both Kelvin-Voigt and standard linear solid model to study their strain behavior. Constant load is applied for the full 5 msec, while the transient load is applied for only 2 msec. The complete simulation parameters are listed in Table 1.

For a Kelvin-Voigt material; the stress-strain equation as a function of time is given by Eq. (1):

1 $$\sigma \left(t\right)=E\epsilon \left(t\right)+ \eta \frac{d\epsilon \left(t\right)}{dt}$$

where $\sigma$ is the applied load in (Pa), $E$ is the modulus of elasticity, $\epsilon$ is the strain in (mm) and $\eta$ is the viscosity in (Pa.sec.).

The strain as a function of time due to Kelvin-Voigt model will follow Eq. (2) when subjected to a sudden stress:

2 $$\epsilon \left(t\right)=\frac{{\sigma }_{o}}{E} (1- {e}^{\frac{-t}{{\tau }_{R}}})$$

where ${\tau }_{R}= \frac{\eta }{E}$ is the retardation time and t is the simulation time in seconds.

Strain as a function of time due to standard linear solid model is given in Eq. (3):

3 $$\epsilon \left(t\right)=\frac{{\sigma }_{o}}{E} \left(1- {e}^{\frac{-t}{{\tau }_{R}}}\right)\left({e}^{\frac{-t}{{\tau }_{R}}}\right)$$

When applying either a constant or a transient load *P*_*applied*_; the total load experienced by the cornea is given by the following Eq. (4):

4 $${P}_{resultant}={P}_{applied} + {P}_{tear film}- IOP$$

where *P*_*tear*_ film is the tear film pressure working in the same direction of the applied load and equals 4.15 mmHg [23], and IOP is the intra-ocular pressure. Tear film pressure is responsible of maintaining corneal tissue constituents in place together.

**Table 1:** Simulation parameters for Kelvin-Voigt and Standard Linear Solid models

Simulation Parameters	Simulation Type	
	*Constant Load*	*Transient Load*
***Excitation Frequency***	0 Hz	1 KHz
***Number of cycles per burst***	----	3
***Excitation window per simulation time***	100%	40%
***Applied Load***	800 Pa	27 KPa
***Simulation Duration***	5 msec	
***Young’s Modulus***	150 KPa	
***Viscosity***	100 Pa.sec.	250 Pa.sec.
***Tear Film Load***	4.15 mmHg ~ 550 Pa	
***Intraocular Pressure (IOP)***	15 mHg ~ 2050 Pa	

### Thermal behavior

#### Temperature rise (TR)

In this section, we will consider a focused ultrasound transducer placed at 28 mm above the corneal tissue of the human eye. A saline cup is placed in the distance between the transducer face till the corneal tissue. Thermal behavior is simulated using the mathematical model of bioheat transfer equation formulated by Pennes [24], [25]. Bioheat equation is used in a former work presented by [24] to study the thermal behavior of human eye due to InfraRed (IR) LASER exposure in ophthalmic refractive procedures. Bioheat transfer for corneal tissue is given by Eq. (5):

5 $${\rho }_{t}{C}_{t}\frac{\partial T}{\partial t}= \nabla \left(k\nabla T\right)+ {W}_{b}{C}_{pb}\left({T}_{a}-T\right)+ {Q}_{m}+H$$

Where ${\rho }_{t}$ represents tissue density, ${C}_{t}$ represents tissue specific heat, ${W}_{b}$ represents the blood perfusion rate, ${C}_{pb}$ represents the specific heat of blood, ${T}_{a}$ represents the arterial blood temperature, $k$ represents the specific tissue thermal conductivity, and $\nabla T$ represents the temperature spatial gradient. The heat generated inside the tissue in response to an external heat source due to temperature rise (TR) is represented by the term *H*. we will model *H* mathematically in the next section. The external heat source in this study is the pressure push due to ultrasonic transient load. The boundary condition equation of the cornea is given by Eq. (6) [24], [26]:

6 $$-k\frac{\partial T}{\partial x}= {h}_{c}\left(T- {T}_{amb}\right)+ \sigma \left({T}^{4}- {T}_{amb}^{4}\right)$$

Where *h*_*c*_ represents the heat transfer coefficient of the cornea. Stefan-Boltzmann constant is represented by σ. The emissivity of the corneal surface is represented by ϵ. ${T}_{amb}$ represents the ambient temperature surrounding the anterior cornea. Equation 6 represents the convection-type thermal exchange between the cornea and the ambient. Bioheat equation parameters’ values are given in Table 2.

**Table 2:** Bioheat parameters’ values for Eqs. (5) & (6) [24]

Quantity	Value
***C***_***t***_	4178
***k***	0.58 W/mK
***W***_***b***_	zero
${\varvec{T}}_{\varvec{a}}$	36.7 °C
***Q***_***m***_	zero
***h***_***c***_	14 W/m^2^ °C
${\varvec{T}}_{\varvec{a}\varvec{m}\varvec{b}}$	30 °C
ϵ	0.975

The temperature rise (TR) rate of change is represented as the sum of all heat effects, such as, the metabolic heat effect, the cooling down / heating up effect due to arterial blood flow, and the net heat conduction-type effect in the human tissue.

#### Pressure wave modelling

Hence, we need to model the ultrasound transient push that is imparted to the corneal tissue mathematically in terms of its pressure function. Then by substituting with this imparted pressure to the medium in Eq. (5), we solve for the temperature rise (TR) for the corneal tissue due to this imparted transient push. The ultrasound pressure push is modelled mathematically for a circular planar piston by Rayleigh-Sommerfeld integral [27], [28] and given by Eq. (7):

7 $$p(z, w)=\rho c{v}_{0}({e}^{\left(ikz\right)}- {e}^{ik\sqrt{({z}^{2}- {a}^{2})}})$$

**Fig. 3:**
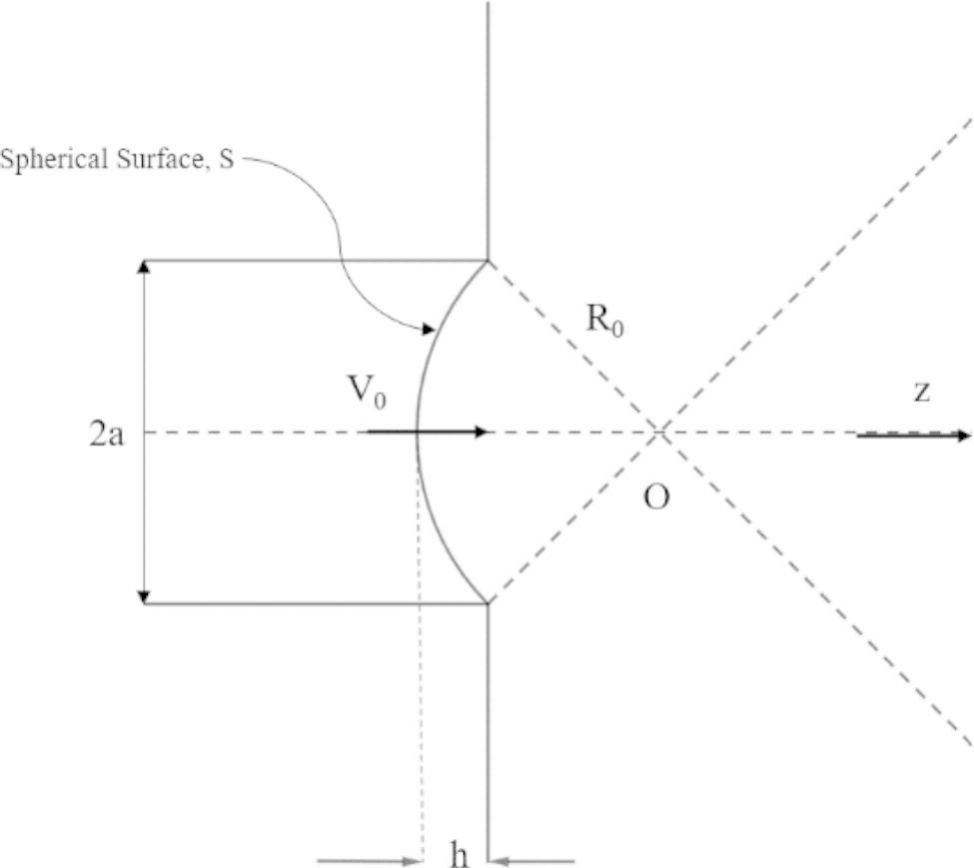
Spherically focused ultrasound transducer

Where a is the radius of the circular piston transducer, v0 is the uniform velocity field over the entire aperture, k is the wave number, and $\rho$ is the density. The equation states that the pressure wave generated is due to two components the direct wave component which is equal to ${e}^{\left(ikz\right)}$, and the edge wave component which is equal to ${e}^{ik\sqrt{({z}^{2}- {a}^{2})}}$. Both components form the on-axis pressure wave. However, for a focused ultrasound transducer the equation can be reduced to Eq. (8). Focused ultrasound transducer can be visualized by means of Fig. 3, where the spherical aperture is denoted by the surface *S*, the circular aperture is of radius *a*, and the spherical surface is of radius *R*_*0*_. Now the integral can be reduced to the following equation:

8 $$p(z, w)=\frac{\rho c{v}_{0}}{{q}_{0}}({e}^{\left(ikz\right)}- {e}^{ik{r}_{e}})$$

Where *q*_*0*_ equals to *1-z/R*_*0*_, and *r*_*e*_ equals to $\sqrt{({(z-h)}^{2}- {a}^{2})}$.

**Fig. 4:**
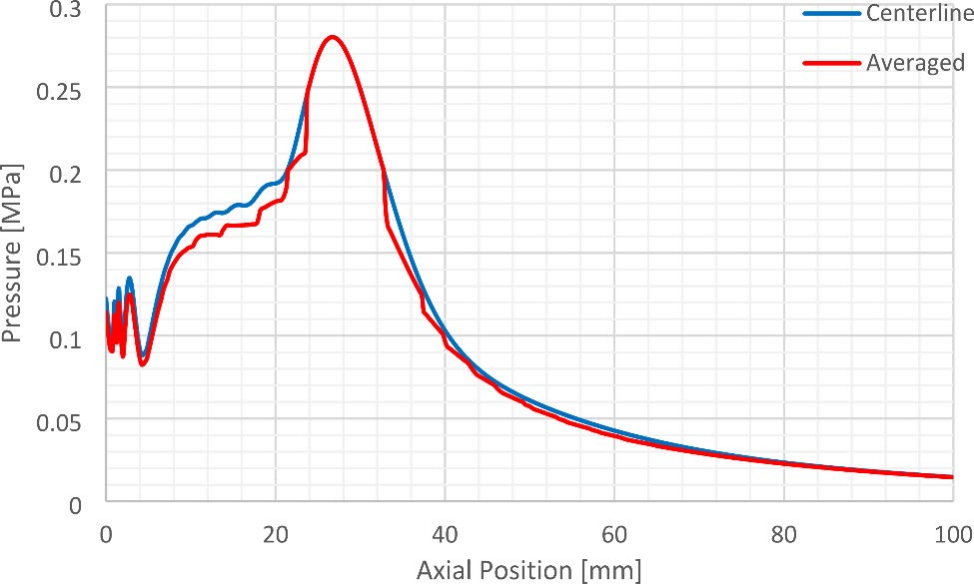
Resulting on-axis pressure wave

The resulting pressure wave that is imparted to the corneal tissue is represented graphically by Fig. 4. The resulting pressure wave is fed into Eq. (5) in order to obtain the temperature rise (TR) due to its effect. The pressure wave distribution presented by Fig. 4 is for both the axial line in front of the transducer and the average pressure wave for lateral positions around the transducer’s center.

## Results and discussion

Simulation is performed using MATLAB program (R2020b) and using a machine of 8 GB RAM and core i5 processor with a best value of execution time of 3.3 msec for transient load scenario and 4 msec for constant load scenario.

### Constant load

For the Kelvin-Voigt model, when a constant load is applied, it starts to deform instantaneously till it reaches an asymptote. The model describes the instantaneous (elastic) deformation due to sudden load, but it fails in describing the viscous damping deformation effect of the viscoelastic material.

**Fig. 5:**
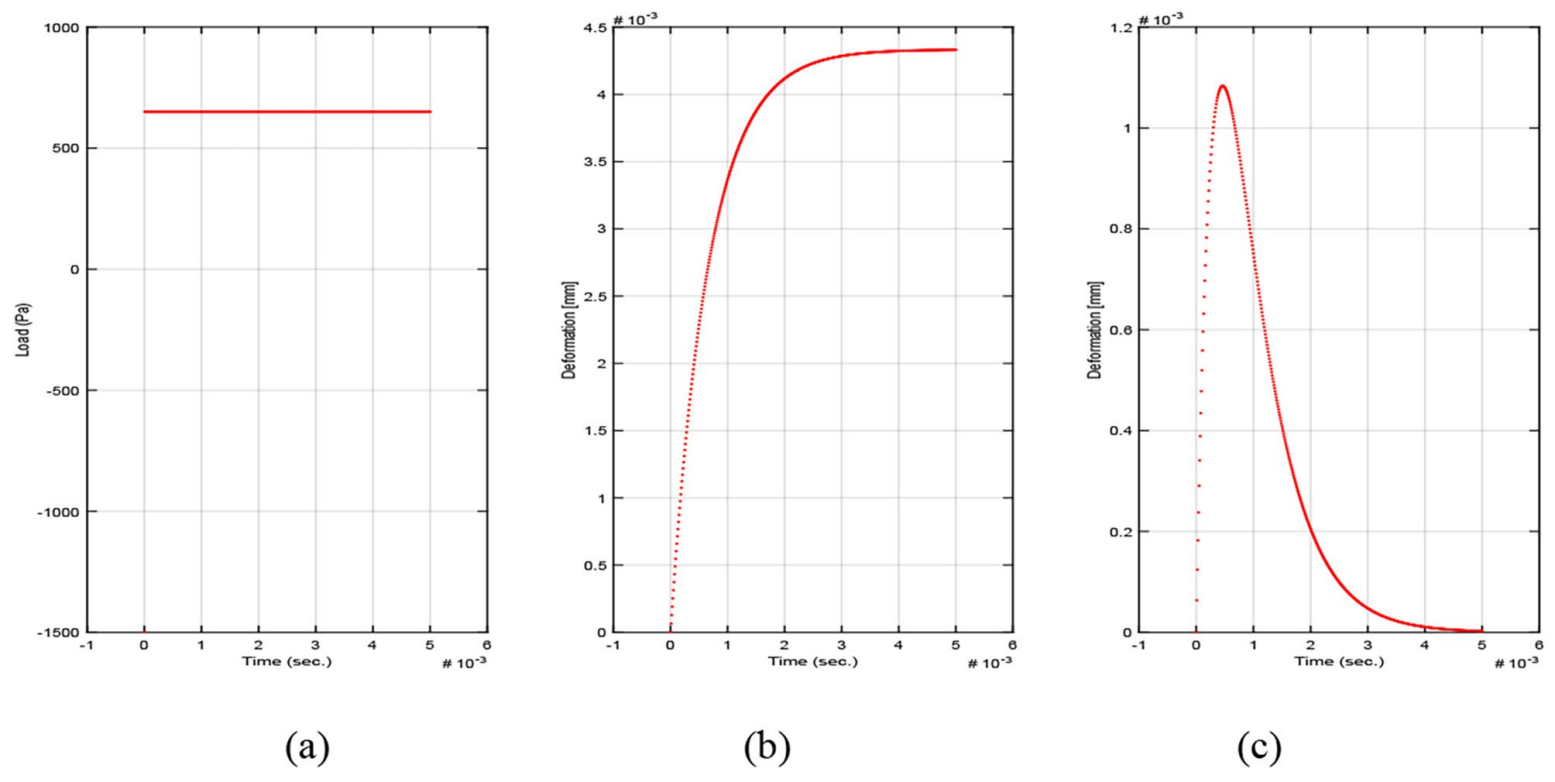
(**a**) Constant load, (**b**) Kelvin-Voigt model time dependent strain behavior and (**c**) Standard linear solid model time dependent strain behavior

The applied constant load is shown in Fig. 5(a). Kelvin-Voigt model time dependent strain (deformation) behavior due to applying constant load is presented in Fig. 5(b). The material does not recover from its deformation, but it continues to creep constantly. The deformation amplitude due to the applied constant load is about 4.4 mm. The material reaches its peak deformation amplitude at 3 msec approximately.

The standard linear solid model is able to describe both the instantaneous (elastic) deformation and the viscous damping deformation of the material. The time dependent strain behavior due to constant load is shown in Fig. 5(c). The deformation amplitude due to this constant load is about 1.1 mm. The material reaches its peak deformation amplitude at 0.45 msec approximately. The material returns to its original state recovering from its deformation.

From the constant load simulation, it is clear that the standard linear solid model is able to describe the corneal tissue viscoelasticity more effectively. This is because it models the material recovery from deformation and because it predicts more reasonable deformation amplitude value compared to Kelvin-Voigt model.

### Transient load

**Fig. 6:**
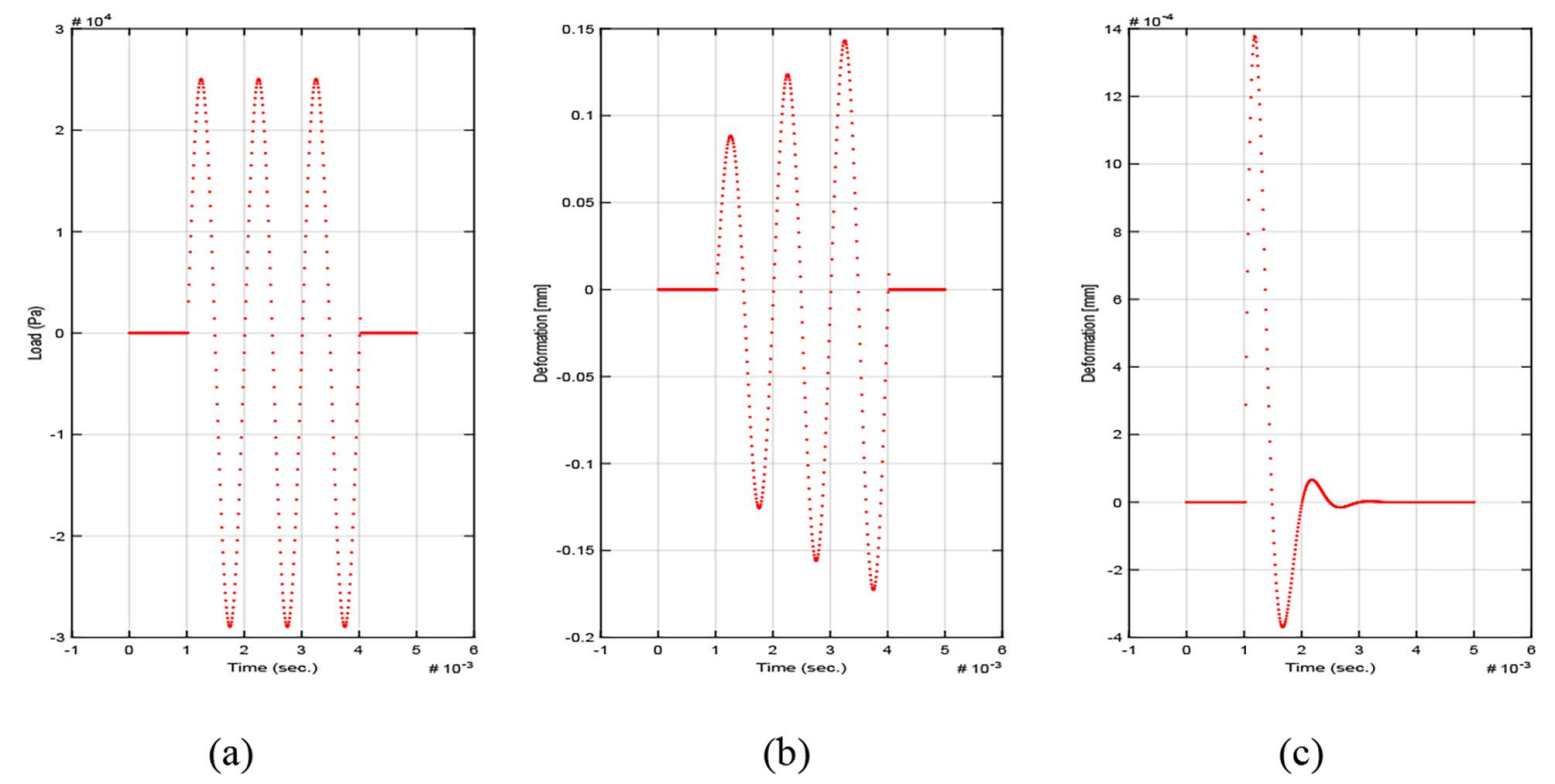
(**a**) Sinusoidal load burst, (**b**) Kelvin-Voigt time dependent strain behavior to transient load and (**c**) standard linear solid model in Kelvin-Voigt time dependent strain behavior to transient load

The applied transient sinusoidal load burst is presented in Fig. 6(a). Kelvin-Voigt model yields a sinusoidal strain behavior that increases with time. The model describes again the instantaneous elastic deformation effectively. However, it fails in describing the viscous damping behavior. The deformation amplitude reaches 0.007 m approximately and continues to increase with each new cycle of excitation which is not logical for corneal tissue. This behavior is presented in Fig. 6(b).

Standard linear solid model behavior is presented in Fig. 6(c). The model predicts the material to have an instantaneous elastic deformation combined with exponential damping for deformation behavior. The deformation amplitude is approximately 1.2 mm which decreases significantly with each cycle of load excitation till reaches original state. The deformation amplitude is reasonable for cornea tissue in response to the applied transient load compared to the deformation amplitude predicted by Kelvin-Voigt model.

In terms of temporal localization, where the deformation is supposed to be highly localized within short temporal duration, the predicted behavior due to Kelvin-Voigt model is not suitable as it does not fulfill the temporal localization for the corneal deformation. On the other hand, standard linear solid model predicts properly the temporal localization of the corneal deformation.

**Fig. 7:**
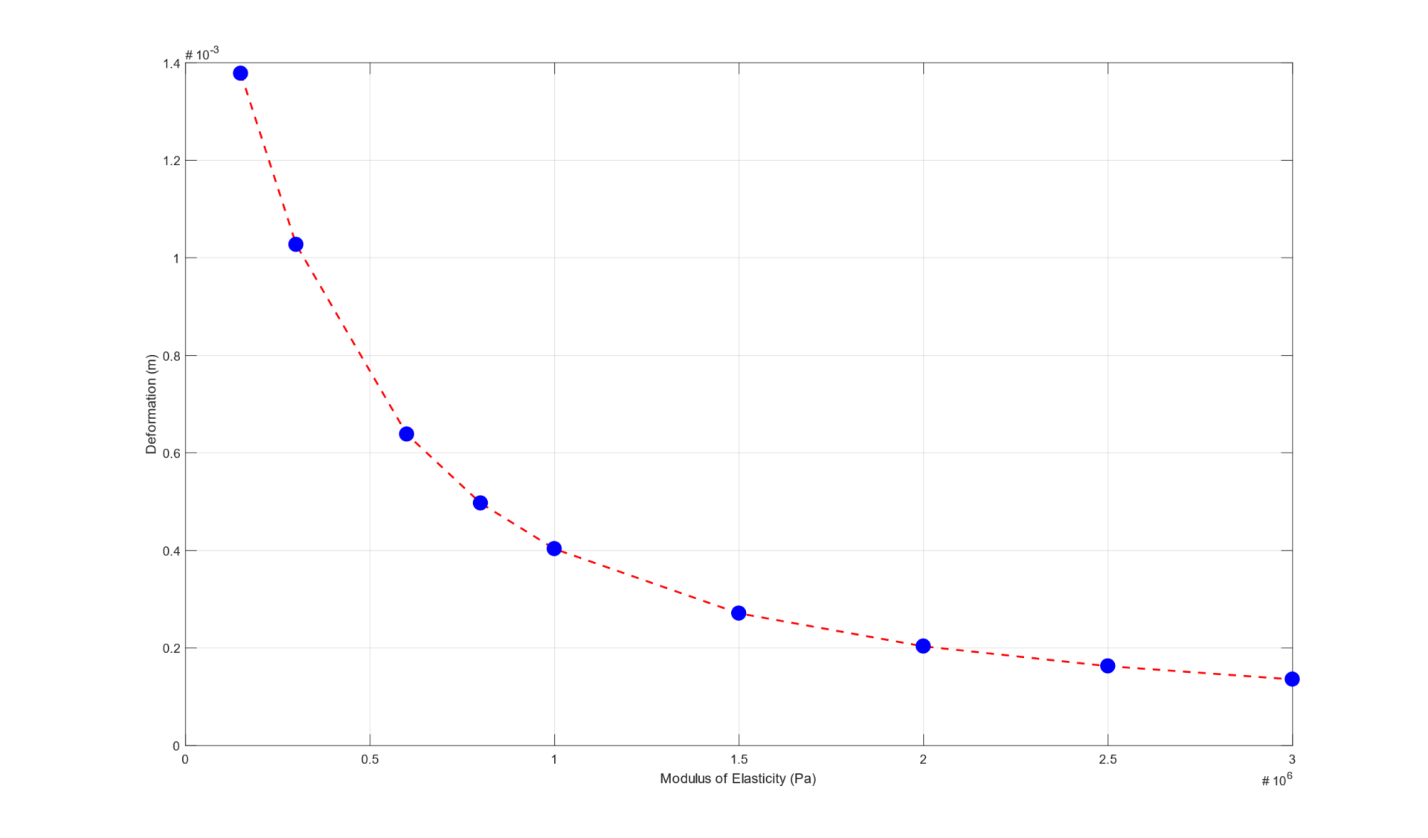
Generalized mathematical model behavior for transient load

The deformation behavior is calculated for different multiple elastic moduli starting from 150KPa to 3 MPa to investigate the mathematical model behavior for a wide range of elastic moduli using SLS model. This is done in order to understand the behavior in both pre-refractive and post-refractive states of the cornea. The complete behavior of the mathematical model for transient load is presented in Fig. 7. It is observed that the behavior is a non-linear behavior.

### Temperature rise (TR)

**Fig. 8:**
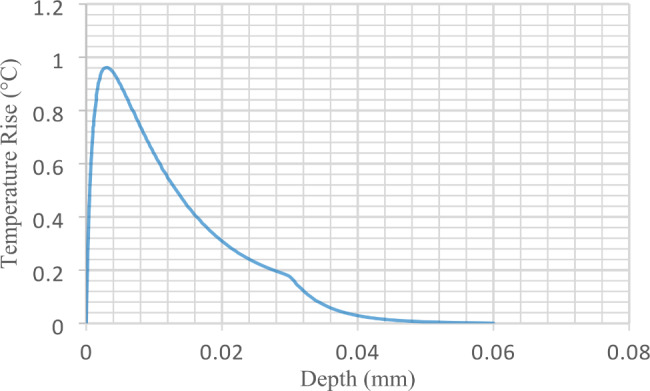
Temperature Rise (TR) in Celsius for the axial line extending from the transducer face till the posterior part of the human eye

The temperature rise (TR) along the axial direction of the transducer is presented by Fig. 8. Temperature rise (TR) is at its maximum near the ultrasound transducer with a ∆T of 0.9 °C. while at the corneal tissue the temperature rise is about 0.17 °C. In addition, corneal thermal behavior is observed to be exponentially decaying with two decaying rates. The first decaying rate is found in the region between the transducer and the surface of the corneal tissue, where this decay rate is slow. The second decaying rate is faster than the first one and found in the region where the corneal tissue is involved and the rest of the human eye structure. Second thermal decaying rate is observed to be faster than the first one because the corneal tissue absorbs much of the imparted thermal energy than the saline cup does. Also, faster decaying rate is due to the fluid-like structures in the human eye such as; aqueous humor in the anterior chamber and the vitreous humor in the posterior chamber, which both act as heat sinks for the imparted thermal energy.

**Fig. 9:**
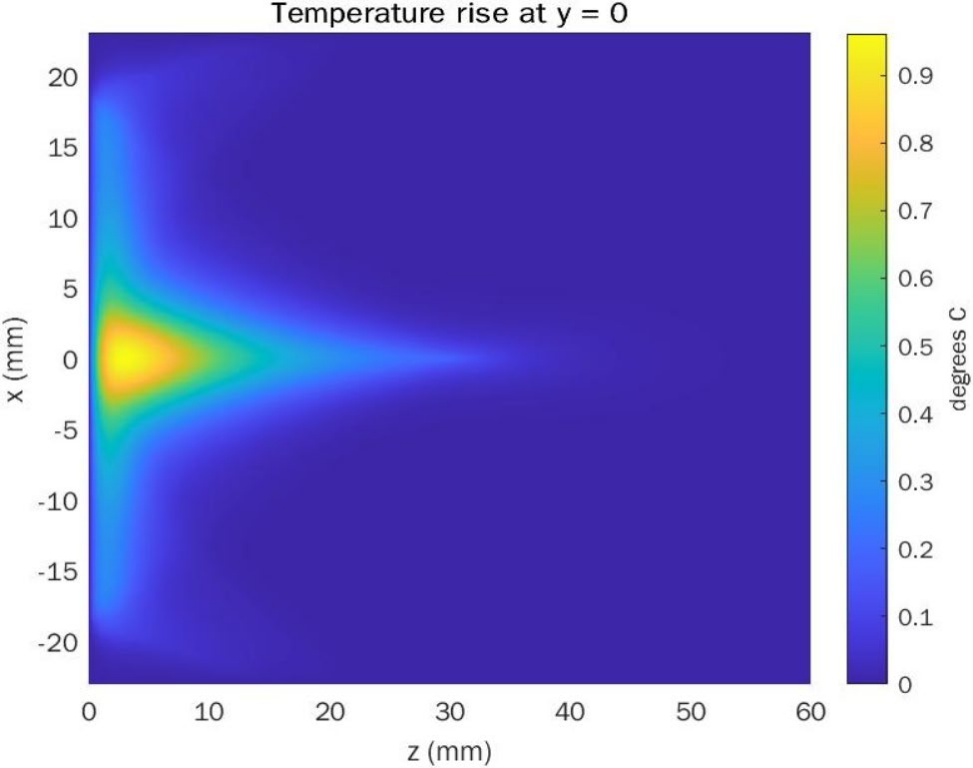
Temperature rise 2D spatial map due to pushing beam

The 2D distribution for the pressure wave generated by the ultrasound transducer is calculated as well and fed to the bioheat equation in order to calculate the 2D temperature rise around both the corneal tissue; anteriorly and posteriorly; and inside the corneal tissue itself. The 2D spatial map for the temperature rise due to the pushing is calculated using bio-heat transfer equation [29] and is presented in Fig. 9.

In terms of temperature rise due to pushing beam, the increase in temperature is concentrated at the proximal area of the transducer forming a delta shaped area of temperature of about 0.9 °C. Hence, the temperature rise is concentrated in front of the transducer directly. From literature, there is a debate about the acceptable temperature rise due to ultrasound waves. As in [30], a temperature rise from 1 to 2 °C is acceptable for all body organs. However, tissue temperature may increase by as much as 10 °C, raising safety concerns even though the acoustic output was still within the Food and Drug Administration’s recommended maximum output exposure level for diagnostic ultrasound [31]. Moreover, The FDA regulates the temperature rise (TR) of tissue to be no more than 6 °C as stated by [32]. In general, the anterior chamber contains a watery-like fluid called aqueous humor, and the posterior chamber contains a gel-like fluid called vitreous humor. Fluids inside the human eye either in the anterior chamber or the posterior chamber act as a heat sink for any generated heat. By consequence, the thermal effect induced due to the acoustic radiation force impulse is reduced by these fluids.

## Conclusion

In this paper, two mathematical models are proposed to simulate corneal viscoelastic behavior. Their performance is compared when applying a constant and transient load respectively. The simulation time is 5 msec for both models. The constant load is applied for the whole simulation time, while the transient load is applied for about 2 msec.

From the obtained results of the two proposed models and with comparison of these two models with each other, it is concluded that standard linear solid model is describing the human corneal behavior in response to constant and transient load more efficiently. This is because of the lack of Kelvin-Voigt model in describing the viscous damping behavior of corneal biomechanics. Also, because the deformation amplitude in Kelvin-Voigt is greater than the reasonable values for the corneal tissue for the same applied load either constantly or transiently.

On the other hand, standard linear solid model predicts a reasonable deformation amplitude for corneal tissue for the same applied load either constantly or transiently. This model also describes the viscous damping behavior of the corneal tissue efficiently.

Also, the thermal behavior of the corneal tissue is calculated using bioheat transfer equation. Temperature rise for corneal due ultrasound pressure push is calculated.

Both the axial and 2D spatial distributions of the temperature rise is calculated for the corneal tissue. Maximum temperature rise (TR) of about 0.9 °C is found to be near to the transducer face. However, the temperature rise for the corneal tissue is found to be about 0.2 °C, which is less the FDA regulations for soft tissue safety.

### Study limitations

We used the fundamental models in biomechanics as a first step in modelling corneal biomechanics. We did not include other advanced models such as Prony and Kevin series with more model parameters for this study. However, we intend to include them in our future improvement of the presented mathematical model to improve the results.

In addition, we calculated the temperature rise for one certain ultrasonic push. Yet, in the future improvement we intend to calculate the temperature rise for a range of ultrasonic pushes in order to determine the maximum push after which the temperature rise inside the cornea will exceed the threshold recommended by FDA. This will guide the manufacturing specifications of the ultrasound probes.

## Data Availability

The datasets presented and analyzed during the current study are available from the corresponding author upon reasonable request.
